# Organizational commitment in the private and public sectors: a regression analysis based on personality traits, subjective wellbeing, organizational orientations, and perceived employment uncertainty in Serbia

**DOI:** 10.3389/fpsyg.2024.1442990

**Published:** 2024-11-01

**Authors:** Dušan Todorović, Petar M. Mitić, Nenad Stojiljković, Mihai Olanescu, Adrian Suciu, Danut Popa

**Affiliations:** ^1^Department of Psychology, Faculty of Philosophy, University of Niš, Niš, Serbia; ^2^Faculty of Sport and Physical Education, University of Niš, Niš, Serbia; ^3^Technical University of Cluj-Napoca, Cluj-Napoca, Romania

**Keywords:** organizational commitment, private and public work organizations, personality traits, employment uncertainty, regression analysis

## Abstract

This study aims to explain the variability in organizational commitment by examining a range of individual and organizational factors. The predictors include personality traits from the HEXACO model, organizational orientations, subjective wellbeing, perceived employment uncertainty, duration of employment, and income satisfaction. The sample consisted of 1,127 employees, with 49.4% from the private sector and 50.6% from the public/state-owned sector. Multiple regression analysis revealed that the models were statistically significant for both sectors. Public sector employees demonstrated higher levels of continuance commitment, likely due to job security, while private sector employees exhibited higher levels of affective and normative commitment. The model accounted for 51.8% of the variance in organizational commitment for public sector employees and 39.2% for private sector employees. These findings underscore the distinct commitment patterns between sectors and emphasize the role of both dispositional and contextual factors in shaping organizational commitment.

## 1 Introduction

Organizational commitment can manifest itself in different forms and impact the organization in several ways. Research indicates that public sector employees often exhibit different levels of organizational commitment compared to their private sector counterparts. For instance, studies have shown that public sector employees tend to have higher normative commitment, which is driven by a sense of duty and obligation, while private sector employees may demonstrate higher affective commitment, which is based on emotional attachment to the organization (Boukamcha, 2022; Markovits et al., 2010; Freire and Azevedo, 2023). However, the picture of organizational commitment is not so simple, as it is necessary to consider the broader context in which this commitment develops. Organizational commitment is shaped not only by the nature of the sector (public or private) but also by individual characteristics and the relationships employees have at work, as well as by the organizational culture and structure (Freire and Azevedo, 2023). The context in which individuals operate, including the interpersonal and organizational dynamics, plays a crucial role in shaping their levels and forms of commitment (Ghumiem and Alawi, 2022; Putri Rahmadani and Winarno, 2023). This complexity is especially pronounced in countries undergoing economic transition. Specific economic, political, and social factors in these countries contribute to a unique organizational landscape (Obschonka and Silbereisen, 2015). The shift from a planned to a market economy, combined with changes in labor laws, privatization processes, and fluctuating job security, significantly influence both public and private sector dynamics (Bogićević Milikić et al., 2012; Pinquart and Silbereisen, 2008). These factors may lead to different patterns of organizational commitment compared to those observed in more stable economies.

Given these distinct challenges and circumstances in transitional economies, this study seeks to further explore the complex interplay between individual, interpersonal, and organizational factors in shaping organizational commitment in both the public and private sectors.

Despite the evident differences between the public and private sectors, there remains a significant gap in research addressing how these distinctions influence organizational commitment, particularly in transitional economies such as Serbia. The rapid development of the private sector over the past decade, driven by the influx of multinational corporations, has reshaped the employment landscape (Bogićević Milikić et al., 2012). Still, it is unclear how these changes have impacted employee commitment across sectors. Existing theoretical frameworks and practical approaches often fail to fully recognize the nuances of these evolving dynamics, highlighting the need for further investigation.

### 1.1 Organizational commitment

Organizational commitment can be determined by the extent of the desires, needs, and obligations that an individual feels toward the organization they work for. Allen and Meyer (1990, 1996) distinguish between three components of organizational commitment: *affective commitment* exists when an employee wants to remain in the organization because of emotional attachment; *normative commitment* stems from feelings of the obligation of the employee to stay in the organization because of the incentives given or benefits offered (salaries and training); *continuance commitment* refers to the notion that there are accumulated benefits that could be lost if one leaves the organization (friends in the workplace, benefits specific to a particular organization). The three-component conceptualization of organizational commitment can be regarded as the dominant model in organizational commitment research (Bentein et al., 2005; Cohen, 2003).

Research shows that people who are committed to the organization to which they belong generate more positive contributions to it than people who are not engaged, which manifested through a firm intention and desire to stay in the organization of which they are already a part, lower absenteeism or turnover intentions and better job performance (Beck and Wilson, 2000; Hausknecht et al., 2009; Metcalfe and Dick, 2001; Lavelle et al., 2009; Reiche, 2008; Mercurio, 2015; Vandenberghe et al., 2004). Employees who are committed to an organization believe that the organization is an excellent place to work, do not search for another workplace in a new organization, have developed positive effects toward the organization, and believe that there are no better alternatives in other organizations that would meet their needs (Mercurio, 2015; Meyer et al., 2002; Perry et al., 2016; Riketta and Van Dick, 2005; Solinger et al., 2008; Todorović et al., 2017a).

Research indicates that employees in the private sector generally exhibit higher levels of organizational commitment than their public sector counterparts. This trend can be attributed to several factors, including differences in job satisfaction, perceived organizational support, and the nature of work environments. A comparative study found that private-sector employees demonstrated significantly higher organizational commitment than their public-sector counterparts, suggesting that the work environment in private institutions fosters greater employee engagement and loyalty (Ali, 2019). This finding is echoed in the work of, those who argue that public-sector organizations could benefit from adopting private-sector practices to enhance employee commitment (Steijn and Leisink, 2006). The authors emphasize that public sector employees often experience lower job satisfaction, which negatively impacts their commitment levels, highlighting the need for effective human resource management practices that can bridge this gap.

Researching organizational commitment in Serbia's public and private sectors is crucial due to its direct impact on individual performance and organizational outcomes (Gajić et al., 2021; Pavlović et al., 2022). In the public sector, organizational commitment is often influenced by perceptions of bureaucratic structures and leadership behavior, where low commitment can lead to absenteeism, reduced productivity, and lower service quality (Janovac et al., 2022; Stankevičiute and Savanevičiene, 2021; Jahan et al., 2022). This is especially important in Serbia, where public sector organizations are seen as hierarchical, affecting employee morale. In the private sector, violations of psychological contracts, particularly regarding job security and career development, can significantly reduce commitment, leading to higher turnover and instability (Shahnawaz and Goswami, 2011). These effects are even more pronounced, given the precarious nature of private sector employment in Serbia. Furthermore, new public management practices in the public sector, aimed at efficiency, may undermine employee commitment if perceived as threatening job security (Oberoi, 2013). Thus, exploring these factors is vital to improve organizational performance across both sectors.

### 1.2 Socioecological approach

Each approach and paradigm of organizational behavior represents partialized analysis (e.g., individual differences paradigm), which comprises a dominant characteristic of organizational psychology (Haslam, 2004). Contemporary theoretical approaches are directed toward the “open systems”—they strive to accept the significance of external factors in a work organization. Therefore, socioecological models place the focus of their interest both on individual behavior and environmental determinants (Bronfenbrenner, 1977, 1979). The socioecological approach represents a theoretical frame, or constellation of theoretical principles, that leads to a fluent explanation of dynamic interrelations of personal and contextual factors (Schulze, 2005). As very few research studies took both dispositional (e.g., personality traits) and contextual variables (e.g., employment uncertainty) into account at the same time during the scientific analysis—implying they primarily present an organization as an open-ecological system—this study in a comprehensive way, using the integrative approach, tries to achieve the prediction of the criteria related to organizational commitment of employees based on a set of predictors, such as personality traits, managerial orientation, subjective wellbeing, and perceived employment insecurity.

Bearing in mind that integrative and open-system approaches could deal with contextual and dispositional aspects simultaneously, it is imperative to consider some variables, such as perceived employment insecurity, level of satisfaction with income, and organizational orientations of employees—from the social sphere in further analysis.

### 1.3 Personality traits

Personality traits have been regarded as significant determinants of individuals' behavior; previous literature refers to personality traits as cognitive (personal values), affective (attitudes), and behavioral patterns (behaviors; Landers and Lounsbury, 2006; Huang et al., 2014; Stanković et al., 2022). Researchers through their several studies have utilized personality traits to identify their influence on employees' behavior (Herrera and Heras-Rosas, 2021; Walumbwa and Schaubroeck, 2009). Extensive data and analyses support the justifiability of the inclusion and measurements of personality traits in organizational psychology (Hogan, 2005; Hough and Oswald, 2008; Ones et al., 2005, 2007). The results of individual studies, whether directly or indirectly, indicate that personality traits are linked to affective commitment at work (Matzler and Renzl, 2007; Naquin and Holton, 2002).

The results of a regression analysis, calculated on a sample of Chinese workers (Cui, 2010) that are culturally and traditionally characterized by a high degree of collectivism, reciprocity in interpersonal relations, and loyalty, have shown that Conscientiousness makes a positive contribution to the explanation of criteria of affective commitment. On the other hand, openness to experiences has also had a statistically significant partial contribution to explaining the total variability of affective commitment, but in a negative sense, whereby a higher level of openness indicates a decrease in affective commitment to work and the organization. Numerous authors (Maertz and Griffeth, 2004; Salgado, 2002; Zimmerman, 2008) suggest a significant association between openness to new experiences and an increased tendency toward abandoning a workplace. In addition, findings confirm the association between individual personality traits and continuance commitment as an aspect of organizational commitment. This primarily refers to traits such as extroversion and neuroticism, while continuance commitment has determined negative correlations (Cui, 2010). Matzler et al. (2011) studied the associations between individual personality traits of employees (Conscientiousness and Conscientiousness) and affective commitment and the readiness of employees to share information with their co-workers, while Ones et al. (2007) confirmed the associations between the aforementioned personality traits and the behavior of employees in the workplace, their attitudes, and work performance.

### 1.4 Subjective wellbeing

Subjective wellbeing can be seen as an individual factor within the socioecological model because it reflects personal evaluations of life satisfaction and emotional wellbeing while being shaped by broader social, cultural, and environmental contexts (Luhmann et al., 2013; Chiu et al., 2013; Garcia et al., 2017; Katić and Ingram, 2017). This positioning emphasizes that, although SWB interacts with external factors, it remains rooted in individual psychological assessments, making it a crucial predictor of behavior and outcomes. Studies show that individuals who characterize their professions and workplaces as having a high level of efficiency, control, and significance and, along with that, a low level of stressors, exhibit higher levels of subjective wellbeing (Christiansen, 2000). The concept of “wellbeing” refers to optimum psychological functioning and experience (Ryan and Deci, 2001). Subjective wellbeing is not a phenomenon that is only significant at the level of an individual; furthermore, it is a fact that happiness and life satisfaction, in addition to economic and social indicators, represent some of the most important indicators of the quality of life in a society. The conclusion is that there is broad theoretical support for the association between the characteristics of a profession, professional engagement, and a subjective evaluation of general subjective wellbeing (Diener and Diener, 2008; Weziak-Bialowolska et al., 2020).

Research consistently demonstrates that higher levels of subjective wellbeing correlate positively with organizational commitment, suggesting that when employees feel good about their psychological state, they are more likely to exhibit commitment to their organization. For instance, a positive relationship was found between subjective wellbeing and organizational commitment among academicians, reinforcing the notion that employees who experience higher subjective wellbeing are more committed to their organizations (Yalçin et al., 2021). Similarly, research studies highlighted that subjective wellbeing is positively related to organizational commitment, indicating that employees who feel happy and comfortable in their work environment are less likely to seek employment elsewhere (Nurrohman and Kustiawan, 2022). Moreover, psychological capital, which encompasses self-efficacy, optimism, hope, and resilience, has been shown to significantly influence organizational commitment. Studies such as those indicate that employees with high psychological capital tend to develop positive attitudes and exhibit higher levels of organizational commitment (Hua, 2020; Simons and Buitendach, 2013).

### 1.5 Organizational orientation

Organizational orientation refers to the overarching attitudes, values, and practices that guide how individuals within an organization perceive their roles, responsibilities, and interactions with others, as well as how they align with the organization's goals. It shapes how employees engage with organizational structures, leadership, and colleagues (McCroskey et al., 2004). Organizational orientation refers to the strategic focus and cultural framework that guide an organization's operations and decision-making processes. It encompasses various dimensions, including market orientation, entrepreneurial orientation, and customer orientation, collectively shaping how organizations interact with their environment and stakeholders (Soomro and Shah, 2019; Kelvin and Joyce, 2019; Ahmed et al., 2018). The importance of organizational orientation lies in its ability to foster an environment conducive to innovation, adaptability, and overall performance. For instance, a strong market orientation has been shown to enhance organizational performance by aligning the organization's strategies with customer needs and market dynamics (Gündogmuş et al., 2024; Kelvin and Joyce, 2019; Yu et al., 2010). Similarly, entrepreneurial orientation, characterized by a willingness to innovate and take risks, has been linked to improved organizational resilience and performance (Asare-Kyire et al., 2023; Maleki and Hajipour, 2020).

The interplay between organizational orientation and commitment is also noteworthy. For instance, organizations that cultivate a strong market orientation tend to foster higher levels of employee commitment by aligning organizational goals with employee values and expectations (Kelvin and Joyce, 2019; Ahmed et al., 2018). Furthermore, perceived organizational support has been identified as a crucial factor influencing organizational commitment and performance, suggesting that employees are more likely to exhibit commitment when they feel supported by their organization (Yu et al., 2010).

### 1.6 Cultural and economic dynamics in transitional economies

When empirical research is conducted in developing and transitional countries such as Serbia, the cultural context in which the research occurs often represents a crucial factor in understanding business and work relations. It is an unavoidable variable in sociopsychological explorations (Larimo and Arslan, 2013), mainly because Europe's Southeastern Europe is a unique mix of cultures, customs, and languages. Each country in this region, including Serbia, is rich in its distinct heritage. Since the 1970's, economic recessions, industrial restructuring, technological “advancements,” and increasingly intense competition have dramatically affected and transformed the very nature of work and employment conditions. The global economy is currently experiencing a severe downturn, with potentially dire economic and social consequences that have a powerful impact on developing countries in transition, such as Serbia and its neighboring countries with similar cultural contexts. Starting in the second half of 2008, a growing number of countries experienced sharp declines in output, which quickly translated into substantial reductions in employment and working hours, and in some cases, unprecedented increases in unemployment [Federal Statistical Office [Statistisches Bundesamt], 2005; Federal Employment Agency [Bundesagentur für Arbeit], 2006; Pinquart et al., 2009; Silbereisen et al., 2006; Silbereisen and Tomasik, 2010].

### 1.7 Job insecurity

Organizations in most global industrial countries have been forced to restructure, let go of their current employees, and “streamline their workforce.” A significant part of the existing literature suggests that perceptions of employment insecurity not only have critical consequences for the actions and attitudes of employees toward the organization (Obschonka and Silbereisen, 2015; Pinquart and Silbereisen, 2004, 2008) and their general psychological wellbeing (Ball, 2009; Bell and Blanchflower, 2009; Córdoba-Doña et al., 2016; Flint et al., 2013; Obschonka and Silbereisen, 2015; Pinquart and Silbereisen, 2008) but also how employees perceive their organization (Hochwarter et al., 2003; Mohr, 2000; Pinquart and Silbereisen, 2008; Sverke and Hellgren, 2001; Tsai and Chan, 2011). Studies indicate that perceived threats and dangers to the nature and existence of the workplace can have adverse outcomes, similar to job loss itself (Marcus and Schuler, 2004; Miller et al., 2008; Pinquart and Silbereisen, 2008; Silbereisen and Tomasik, 2010). All this is congruent with the basic assumption underlying research in the field of psychology of stress, which is that the anticipation of stressful events represents an equally essential and strong, maybe even the strongest, source of anxiety; even more so than a real, actual event (Lazarus and Folkman, 1984).

Research consistently shows a negative correlation between job insecurity and organizational commitment. For example, Praptiningstyas et al. (2021) found that job insecurity negatively impacts performance and organizational commitment. Vujičić et al. (2014) reported that employees experiencing job insecurity exhibit lower job satisfaction and commitment. Similarly, Zyl et al. (2013) found that cognitive and affective dimensions of job insecurity increase job-related stress and reduce commitment. Sora et al. (2011) further emphasize that job insecurity decreases life satisfaction, job satisfaction, and organizational commitment, reinforcing its role as a significant stressor. Öztürk et al. (2017) found that affective commitment can buffer adverse emotional reactions to job insecurity, suggesting the value of a supportive environment. Finally, Hsieh and Kao (2021) highlighted how job insecurity leads to negative work environment perceptions, affecting engagement and satisfaction, a point further reinforced by Vera Andriyanti and Suardana (2023) and Bohle et al. (2018) regarding the importance of organizational support in maintaining commitment.

### 1.8 Duration of employment and satisfaction with income

The relationship between the duration of employment and organizational commitment is well-documented, with longer tenure often correlating with stronger emotional ties and a more profound sense of belonging to the organization (Bashir and Long, 2015). Organizational support practices, such as training and development, enhance affective commitment, especially for long-term employees, as their extended time in the organization allows for positive experiences and support (Arasanmi and Krishna, 2019; Tansky and Cohen, 2001). This is particularly evident among long-term employees who have a vested interest in the organization's success (Onur, 2024). Finally, effective human resource management practices that promote career development and employability are strongly linked to higher levels of organizational commitment, particularly for long-tenured employees, as these practices reinforce perceptions of career progression and organizational investment (Ahmad et al., 2023; Hussain et al., 2022).

The relationship between income satisfaction and organizational commitment is complex, with changes in income often more impactful than static levels. Research highlights that perceptions of income changes significantly influence job satisfaction, which in turn affects organizational commitment (Wang et al., 2023). Job satisfaction is a mediator, enhancing emotional attachment and loyalty to the organization (Fu, 2023; Yang et al., 2019). Studies also show that fair and supportive work environments foster commitment through improved job satisfaction (Sutiyoso, 2016). Satisfaction with factors such as pay, promotion opportunities, and leadership behaviors is positively correlated with organizational commitment (Araya and Ma, 2016; Mohapatra et al., 2019). Moreover, organizational commitment helps reduce turnover intentions and boost employee performance (Bachri and Solekah, 2021; Hasan et al., 2019).

### 1.9 Current study

Building upon the existing literature and considering the unique cultural and economic dynamics of transitional economies such as Serbia, the current study explores how individual, interpersonal, and organizational factors collectively influence organizational commitment among employees in both the private and public sectors. Utilizing a socioecological framework, the study addresses the gap in previous research that often overlooks the simultaneous impact of dispositional and contextual variables on organizational commitment. This approach acknowledges the organization as an open system, influenced by both internal characteristics of employees and external environmental factors.

**Figure 1:**
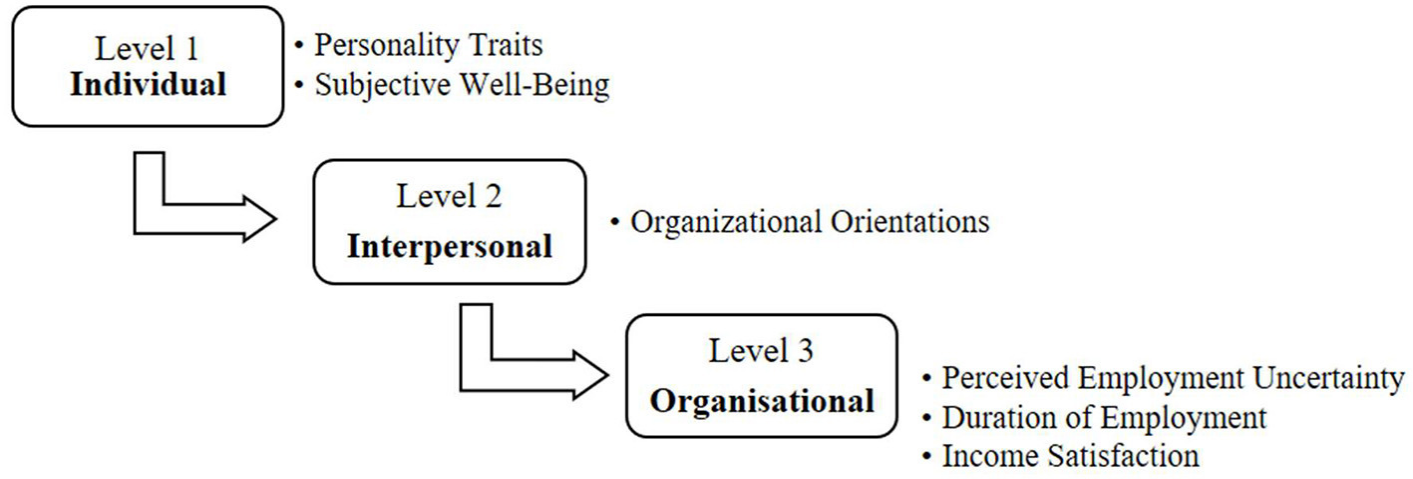
The socioecological model tested in a multiple regression analysis.

The research design is primarily abductive, adopting an exploratory approach to understand the complex interactions between individual, interpersonal, and organizational factors influencing organizational commitment. The study did not include specific hypotheses, as the aim was to allow for a more comprehensive examination of these dynamics. The primary objective is to explore how these factors collectively impact organizational commitment among employees in both private and public sectors in Serbia, utilizing a socioecological framework (Figure 1).

The primary goal of this study is to enhance our understanding of how these factors interact in the context of a transitional economy, where economic instability, cultural nuances, and organizational restructuring significantly affect employee attitudes and behaviors. Focusing on Serbia provides a unique opportunity to examine these dynamics in a setting characterized by rapid economic and social changes. The findings offer valuable insights for both academia and practitioners, informing strategies to foster organizational commitment and improve employee retention and performance in similar economic contexts.

## 2 Methods

### 2.1 Participants

The research sample consisted of 1,127 respondents from Serbia. Only seven respondents were excluded from the analysis as they were identified as outliers based on the Mahalanobis distance calculation. All other responses were included, as participants provided complete and valid data sets, ensuring the integrity of the analysis. By gender, 50.5% were female and 49.5% were male. Age ranges from 20 to 65 years, with an average of 39 years. 1.8% of participants have a Ph. D., 24.3% hold an MA or MSc, 12.4% have a bachelor's degree and 56.1% possess a high school diploma, while 4.2% have a lower level of education, such as an elementary school degree. 81.6% of participants came from urban areas, while 18.4% came from rural areas (villages or suburban regions). Duration of employment (years of service in an organization) varied from 1 to 40 years. 34.4% of employees work in the industry (in manufacturing roles), 30.4% hold administrative roles, and 35.1% are in service jobs. Participants were recruited using purposive sampling, specifically aiming to include a diverse group of employees from different organizational backgrounds. The selection process was designed to ensure representation from both sectors, reflecting a range of job roles, from administrative and service jobs to industrial and manufacturing roles. Participants were approached directly through their organizations, which agreed to facilitate data collection, and they voluntarily completed the assessment instruments. The advantage of purposive sampling is the opportunity to create justification to generalize from the sample, considering that it is a specific category of employees. All research procedures with respondents were conducted in the second and third quartal of 2021 in Serbia's private-owned and state-owned work organizations. All respondents came from various work organizations, such as the food industry, recycling industry, public services, the medical sector (hospitals, polyclinics, and ambulances), military services, local administration–clerical institutions, 557 (49.4%) of whom were employed in the private sector and 570 (50.6%) were from public/state-owned sector. Data collection process: employees from various work organizations voluntarily responded individually to a set of printed instruments. All participants who chose to take part completed the assessment instruments fully. It took ~45 min due to the number of items in the psychological assessment tools. Data were collected individually, capturing demographic information, employment characteristics, and responses to psychological assessment tools. There was no data collection at higher organizational levels or across multiple hierarchical tiers within the same organization.

### 2.2 Instruments

To measure the intensity of organizational commitment Allen–Meyer's organizational commitment questionnaire was used (the Organizational Commitment Scale, Allen and Meyer, 1990, 1996; Meyer and Allen, 1991), which has the following subscales, that is, it measures the following aspects of organizational commitment: affective commitment, continuance commitment, and normative commitment. Confirmatory factor analysis indicated that the validities and reliabilities of the respective constructs of organizational commitment, namely, affective commitment, continuance commitment, and normative commitment when taken individually, were satisfactory. However, the three constructs were highly intercorrelated. Hence, it was recommended that researchers on organizational commitment continue to use the model confidently (Allen and Meyer, 1996; Cohen, 1996; Mugizi et al., 2016).

The instrument used to determine personality traits was the Honesty–Humility, Emotionality, Extraversion, Agreeableness, Conscientiousness, and Openness to Experience Personality Inventory-Revised (HEXACO-PI-R) model (Lee and Ashton, 2006), which consists of 60 items in total. The dimensions represented were as follows: Honesty, emotionality, extraversion, agreeableness, conscientiousness, and openness.

An organizational orientation questionnaire was used to measure the intensity of organizational orientation (McCroskey et al., 2004).

General Subjective wellbeing was measured using the Concise Scale of Subjective Wellbeing (Jovanović and Novović, 2008).

To measure perceived employment uncertainty, a questionnaire was designed for the purpose of this study, one based on a scale for measuring perceived requirements related to the work context, and, thus, perceived risk of job loss or change of workplace (Silbereisen et al., 2006). The complete questionnaire created by these authors consists of a great number of smaller scales; however, in our study, we only used the scale whose items directly refer to perceived employment uncertainly (perceived risk of job loss, perceived lack of workplaces, and opportunities for employment), as well as a perceived decrease in work conditions (more frequent overtime, working the night shift), a lack of time for hobbies and leisure activities, and thus a more intense need for financial support from family or friends.

The reliability of the instruments used has been proven through the research process: the value of Cronbach's α for the questionnaire used to measure organizational commitment is 0.861 (affective α = 0.71; continuance α = 0.70; normative α = 0.68); the reliability of the HECACO-PI-R questionnaire obtained by calculating Cronbach's α is 0.781, and the dimensions represented were as follows: Honesty (α = 0.69), emotionality (α = 0.72), extraversion (α = 0.67), Agreeableness (α = 0.61), conscientiousness (α = 0.75), and openness (α = 0.73); the reliability for the questionnaire used to measure subjective wellbeing is 0.880; for the questionnaire used to measure employment uncertainty it is 0.670; Cronbach's α for the questionnaires used to measure organizational orientation ranges from 0.824 for the questionnaire used to measures the expression of ambivalent orientation to 0.790 for the questionnaire used to measure indifferent organizational orientation; Cronbach's alpha for the questionnaire used to measure orientation toward advancement in the hierarchy of the organization is 0.811. From these findings, it can be concluded that all the questionnaires used have satisfactory reliability. Pallant (2001) states Cronbach's α value above 0.6 is considered high reliability and acceptable index (Nunnally and Bernstein, 1994). The value of Cronbach's α < 0.6 is considered low.

Control variables, such as age, gender, and duration of employment, were included due to their established relationships with organizational commitment. Research indicates that age and tenure are often linked to higher commitment levels, while gender differences can affect how commitment is perceived and expressed (McCroskey et al., 2004; Mercurio, 2015). These controls helped isolate the effects of the main predictors.

### 2.3 Data analysis

Descriptive statistics were calculated for all variables, including mean values, standard deviations, skewness, and kurtosis. Pearson correlations were calculated to explore the relationships between variables. Multiple linear regression analysis was conducted to examine the contribution of individual and contextual predictors on organizational commitment, with all predictors entered simultaneously in a single block. Mahalanobis distance calculations were performed to identify potential outliers, and standard procedures were followed to ensure transparent and accurate reporting. No control group was used; the study analyzed data from a single sample of employees across various sectors. Regression analysis is a powerful statistical tool widely used across scientific disciplines to understand relationships between variables and make predictions (Fox, 2015). In the regression analyses, we controlled for gender by ensuring a balanced distribution within the sample (50.5% females; 49.5% males), which mitigated the need for explicit control of gender effects. Additionally, length of employment and income satisfaction, two relevant demographic variables, were included as predictors in the regression models, making it unnecessary to control for them separately. Following the recommendation of Bernerth and Aguinis (2016), control variables were included only if there was a clear statistical or theoretical justification. While other relevant variables (e.g., education and place of residence) were measured, they were not included in the analysis due to their categorical nature and unequal distribution. These variables do not represent a continuous spectrum, and their uneven distribution makes them less suitable for control within regression analysis. Discriminant analysis was used to distinguish between private and public sector employees based on their levels of organizational commitment. A significance level of *p* ≤ 0.05 was used for all statistical tests. For all corrections made during the revision process, several procedures were applied. This included recalculating regression results to ensure accuracy and robustness, creating new tables to represent better findings, and referencing similar studies published in Frontiers scientific journal to provide a stronger contextual foundation. Additionally, more recent literature was searched thoroughly to integrate the latest insights and enhance the theoretical background. These steps were taken to address reviewer feedback and improve the overall quality and rigor of the study.

## 3 Results

A predictive model was developed using multiple regression analysis to assess the potential for predicting organizational commitment among employees, with all variables included in a single set of predictors. The study aimed to determine the extent to which organizational commitment can be explained by a set of predictor variables, including personality traits from the HEXACO model, organizational orientation (hierarchy-focused, ambivalent, and indifferent), subjective wellbeing, perceived employment uncertainty, duration of employment, and income satisfaction.

The initial results from the correlation analysis are presented in Table 1. Organizational commitment, particularly its affective and normative aspects, shows a positive correlation with honesty and agreeableness, and, to a lesser extent, with emotionality, extraversion, and conscientiousness. Additionally, there are positive correlations with subjective wellbeing, employment duration, income satisfaction, and an organizational orientation focused on advancement within the hierarchy. Conversely, employees with pronounced ambivalent or indifferent organizational orientations exhibit lower levels of organizational commitment, especially in its affective aspect. Perceived employment uncertainty also weakens organizational commitment.

**Table 1:** Descriptive and correlation analysis results.

	**AOC**	**COC**	**NOC**	**OC**	**HH**	**E**	**Ex**	**A**	**C**	**O**	**SWB**	**HOO**	**AOO**	**IOO**	**PEU**	**ED**	**IS**
COC	0.379^**^	-															
NOC	0.768^**^	0.502^**^	-														
OC	0.869^**^	0.682^**^	0.943^**^	-													
HH	0.251^**^	−0.008	0.222^**^	0.205^**^	–												
E	0.032	0.152^**^	0.120^**^	0.118^**^	−0.046	–											
Ex	0.185^**^	−0.081^**^	0.135^**^	0.115^**^	0.155^**^	−0.144^**^	-										
A	0.216^**^	0.116^**^	0.277^**^	0.256^**^	0.247^**^	0.007	−0.012	-									
C	0.228^**^	−0.033	0.179^**^	0.166^**^	0.336^**^	−0.030	0.390^**^	0.174^**^	-								
O	−0.012	−0.185^**^	−0.055	−0.085^**^	0.038	−0.041	0.246^**^	−0.114^**^	0.195^**^	-							
SWB	0.271^**^	−0.014	0.236^**^	0.217^**^	0.138^**^	−0.035	0.448^**^	0.140^**^	0.296^**^	0.191^**^	-						
HOO	0.256^**^	−0.027	0.210^**^	0.195^**^	0.070^*^	−0.001	0.454^**^	0.015	0.422^**^	0.116^**^	0.414^**^	-					
AOO	−0.427^**^	0.108^**^	−0.281^**^	−0.271^**^	−0.419^**^	0.030	−0.277^**^	−0.243^**^	−0.481^**^	−0.099^**^	−0.299^**^	−0.357^**^	-				
IOO	−0.340^**^	0.137^**^	−0.221^**^	−0.200^**^	−0.344^**^	0.100^**^	−0.233^**^	−0.101^**^	−0.367^**^	−0.145^**^	−0.106^**^	−0.247^**^	0.512^**^	-			
PEU	−0.210^**^	0.203^**^	−0.108^**^	−0.075^*^	−0.118^**^	0.116^**^	−0.216^**^	0.039	−0.211^**^	−0.153^**^	−0.214^**^	−0.233^**^	0.385^**^	0.366^**^	-		
ED	0.217^**^	0.301^**^	0.244^**^	0.291^**^	0.001	0.061^*^	−0.209^**^	0.120^**^	−0.061^*^	−0.109^**^	−0.216^**^	−0.305^**^	0.058	−0.030	0.122^**^	-	
IS	0.119^**^	−0.028	0.124^**^	0.100^**^	0.034	0.023	0.133^**^	0.032	0.088^**^	0.099^**^	0.136^**^	0.135^**^	−0.146^**^	−0.146^**^	−0.274^**^	−0.093^**^	-
*M*	15.021	12.838	2.298	48.157	37.309	31.515	34.612	31.967	38.042	32.666	31.925	39.095	21.727	25.240	15.140	14.630	1.977
*SD*	3.681	2.906	5.466	1.336	6.324	6.281	5.473	5.825	5.925	7.215	5.671	5.862	8.004	6.732	4.466	1.614	0.747
*Sk*	−0.612	−0.269	−0.236	−0.422	−0.484	−0.067	−0.284	−0.300	−0.410	−0.066	−0.723	−0.408	0.712	−0.062	−0.001	0.527	0.238
*Ku*	−0.030	0.298	−0.472	−0.027	0.158	0.023	−0.138	0.347	0.190	−0.404	0.488	0.108	0.117	−0.040	−0.380	−0.831	−0.639

HH, Honesty-Humility; E, Emotionality; Ex, Extraversion; A, Agreeableness; C, Conscientiousness; O, Openness to Experiences; SWB, Subjective Wellbeing; HOO, Organizational Orientation: Hierarchy-focused; AOO, Organizational Orientation: Ambivalent; IOO, Organizational Orientation: Indifferent; PEU, Perceived Employment Uncertainty; ED, Duration of Employment; IS, Degree of Income Satisfaction; AOC, Affective Organizational Commitment; COC, Continuance Organizational Commitment; NOC, Normative Organizational Commitment; OC, Organizational Commitment (Overall); M, Mean; SD, Standard Deviation; Sk, Skewness, Ku, Kurtosis.

^*^p < 0.05; ^**^p < 0.01.

However, it is essential to note that all correlations, except for affective commitment with ambivalent and indifferent organizational orientations, are of low intensity (*r* < 0.30), strongly indicating that there are no signs of multicollinearity in subsequent predictions (Allison, 1999). Additionally, we conducted specific tests for multicollinearity, confirming that the relevant indicators were within acceptable levels (Field, 2009). The tolerance scores for each predictor exceeded 0.20, and the variance inflation factor (VIF) values were below 1 (Table 2).

**Table 2:** Regression results for overall organizational commitment in full, private, and public sector samples.

	**Full sample**					**Private sector**					**Public sector**				
	***B***	**SE**	β	**95% CI LB**	**95% CI UB**	***B***	**SE**	β	**95% CI LB**	**95% CI UB**	***B***	***SE***	β	**95% CI LB**	**95% CI UB**
Con.	7.293	4.836		−2.197	16.783	19.257	7.627		4.270	34.243	2.060	6.224		−1.166	14.286
HH	0.171	0.050	0.105	0.072	0.270	0.225	0.070	0.141	0.087	0.363	0.072	0.072	0.043	−0.070	0.214
E	0.178	0.044	0.108	0.091	0.265	0.078	0.062	0.046	−0.044	0.199	0.264	0.064	0.161	0.138	0.389
Ex	0.034	0.062	0.018	−0.088	0.157	−0.043	0.097	−0.021	−0.233	0.148	0.009	0.082	0.005	−0.152	0.169
A	0.261	0.051	0.148	0.162	0.361	0.134	0.073	0.074	−0.009	0.277	0.372	0.070	0.217	0.235	0.509
C	−0.106	0.058	−0.061	−0.220	0.008	−0.284	0.082	−0.155	−0.446	−0.122	0.073	0.082	0.044	−0.088	0.233
O	−0.133	0.041	−0.092	−0.213	−0.054	−0.122	0.055	−0.085	−0.230	−0.015	−0.156	0.060	−0.107	−0.274	−0.038
SWB	0.312	0.058	0.171	0.198	0.426	0.416	0.084	0.225	0.250	0.581	0.236	0.080	0.130	0.078	0.393
HOO	0.361	0.060	0.203	0.244	0.479	0.482	0.087	0.263	0.311	0.652	0.220	0.082	0.125	0.058	0.382
AOO	−0.073	0.048	−0.057	−0.166	0.020	−0.233	0.073	−0.173	−0.376	−0.090	0.045	0.064	0.036	−0.081	0.171
IOO	−0.119	0.050	−0.079	−0.218	−0.021	−0.192	0.066	−0.135	−0.322	−0.063	0.010	0.077	0.006	−0.141	0.161
PEU	0.064	0.071	0.028	−0.076	0.203	0.057	0.094	0.025	−0.128	0.243	0.042	0.105	0.018	−0.165	0.249
ED	0.353	0.028	0.361	0.298	0.407	0.380	0.042	0.359	0.297	0.462	0.321	0.037	0.350	0.249	0.394
IS	1.000	0.381	0.072	0.252	1.748	0.643	0.528	0.045	−0.394	1.680	1.151	0.538	0.086	0.094	2.207
*F*	32.013					24.210					13.677				
*df*	13,1,036					13,489					13,533				
*p*	0.000					0.000					0.000				
*R*	0.535					0.626					0.500				
*R* ^2^	0.287					0.392					0.250				

HH, Honesty-Humility; E, Emotionality; Ex, Extraversion; A, Agreeableness; C, Conscientiousness; O, Openness to Experiences; SWB, Subjective Wellbeing; HOO, Organizational Orientation: Hierarchy-focused; AOO, Organizational Orientation: Ambivalent; IOO, Organizational Orientation: Indifferent; PEU, Perceived Employment Uncertainty; ED, Duration of Employment; IS, Degree of Income Satisfaction; B, unstandardized regression coefficient; SE, standard error; β, standardized regression coefficient; 95% CI LB, lower bound of the 95% confidence interval; 95% CI UB, upper bound of the 95% confidence interval.

For the total sample, the model explained 28.7% of the variance in overall organizational commitment (*R*^2^ = 0.287), and the model was statistically significant, *F*_(13, 1, 036)_ = 32.013, *p* < 0.001. In the private sector sample, the model accounted for 39.2% of the variance in organizational commitment (*R*^2^ = 0.392), with a statistically significant result, *F*_(13, 489)_ = 24.210, *p* < 0.001. For the public sector sample, the model explained 25.0% of the variance in organizational commitment (*R*^2^ = 0.250), and the model was also statistically significant, *F*_(13, 533)_ = 13.677, *p* < 0.001.

For the total sample, honesty–humility (β = 0.105), emotionality (β = 0.108), agreeableness (β = 0.148), subjective wellbeing (β = 0.171), and organizational orientation: hierarchy-focused (β = 0.203) were positively associated with organizational commitment. In the private sector subsample, honesty–humility (β = 0.141), subjective wellbeing (β = 0.225), and organizational orientation: hierarchy-focused (β = 0.263) were positively associated with organizational commitment, while organizational orientation: ambivalent (β = −0.173) showed a negative relationship with organizational commitment. For the public sector subsample, emotionality (β = 0.161), agreeableness (β = 0.217), subjective wellbeing (β = 0.130), and organizational orientation: hierarchy-focused (β = 0.125) were significant positive predictors of organizational commitment.

Table 3 presents the regression results for affective organizational commitment across the full sample and the private and public sector subsamples, highlighting the explained variance and significant predictors in each group. For the total sample, the model explained 32.9% of the variance in affective organizational commitment (*R*^2^ = 0.329), with a statistically significant result, *F*_(13, 1, 036)_ = 39.059, *p* < 0.001. In the private sector subsample, the model accounted for 43.8% of the variance in affective organizational commitment (*R*^2^ = 0.438), with a statistically significant result, *F*_(13, 489)_ = 29.322, *p* < 0.001. For the public sector subsample, the model explained 25.8% of the variance in affective organizational commitment (*R*^2^ = 0.258), and the model was statistically significant, *F*_(13, 533)_ = 14.259, *p* < 0.001.

**Table 3:** Regression results for affective organizational commitment in full, private, and public sector sample.

	**Full sample**					**Private sector**					**Public sector**				
	***B***	**SE**	β	**95% CI LB**	**95% CI UB**	***B***	**SE**	β	**95% CI LB**	**95% CI UB**	***B***	**SE**	β	**95% CI LB**	**95% CI UB**
Con.	7.936	1.667		4.665	11.207	9.377	2.686		4.100	14.654	7.646	2.132		3.458	11.834
HH	0.042	0.017	0.072	0.008	0.076	0.081	0.025	0.138	0.032	0.129	−0.019	0.025	−0.034	−0.068	0.029
E	0.026	0.015	0.044	−0.004	0.056	0.017	0.022	0.028	−0.026	0.060	0.042	0.022	0.074	−0.001	0.085
Ex	0.011	0.021	0.017	−0.031	0.053	0.016	0.034	0.022	−0.051	0.083	−0.015	0.028	−0.024	−0.070	0.040
A	0.056	0.017	0.089	0.021	0.090	0.033	0.026	0.049	−0.018	0.083	0.075	0.024	0.128	0.028	0.122
C	−0.055	0.020	−0.089	−0.094	−0.015	−0.126	0.029	−0.187	−0.183	−0.069	0.028	0.028	0.050	−0.027	0.083
O	−0.027	0.014	−0.053	−0.055	0.000	−0.018	0.019	−0.034	−0.056	0.020	−0.046	0.021	−0.092	−0.086	−0.006
SWB	0.118	0.020	0.182	0.079	0.158	0.114	0.030	0.168	0.056	0.172	0.129	0.028	0.206	0.074	0.183
HOO	0.108	0.021	0.171	0.068	0.149	0.145	0.031	0.216	0.085	0.205	0.075	0.028	0.124	0.020	0.131
AOO	−0.098	0.016	−0.215	−0.131	−0.066	−0.139	0.026	−0.282	−0.190	−0.089	−0.062	0.022	−0.144	−0.105	−0.019
IOO	−0.074	0.017	−0.137	−0.108	−0.040	−0.090	0.023	−0.174	−0.136	−0.045	−0.043	0.026	−0.076	−0.095	0.009
PEU	−0.032	0.024	−0.039	−0.080	0.016	−0.025	0.033	−0.030	−0.090	0.041	−0.058	0.036	−0.071	−0.128	0.013
ED	0.106	0.010	0.306	0.088	0.125	0.110	0.015	0.283	0.081	0.139	0.098	0.013	0.311	0.074	0.123
IS	0.201	0.131	0.041	−0.057	0.459	0.230	0.186	0.044	−0.135	0.596	0.106	0.184	0.023	−0.255	0.468
*F*	39.059					29.322					14.259				
*df*	13,1,036					13,489					13,533				
*p*	0.000					0.000					0.000				
*R*	0.574					0.662					0.508				
*R^2^*	0.329					0.438					0.258				

HH, Honesty-Humility; E, Emotionality; Ex, Extraversion; A, Agreeableness; C, Conscientiousness; O, Openness to Experiences; SWB, Subjective Wellbeing; HOO, Organizational Orientation: Hierarchy-focused; AOO, Organizational Orientation: Ambivalent; IOO, Organizational Orientation: Indifferent; PEU, Perceived Employment Uncertainty; ED, Duration of Employment; IS, Degree of Income Satisfaction; B, unstandardized regression coefficient; SE, standard error; β, standardized regression coefficient; 95% CI LB, lower bound of the 95% confidence interval; 95% CI UB, upper bound of the 95% confidence interval.

For the full sample, honesty–humility (β = 0.072), agreeableness (β = 0.089), subjective wellbeing (β = 0.182), organizational orientation: hierarchy-focused (β = 0.171), and duration of employment (β = 0.306) were positively associated with affective organizational commitment, while organizational orientation: ambivalent (β = −0.215) and organizational orientation: Indifferent (β = −0.137) were negatively associated. In the private sector subsample, honesty–humility (β = 0.138), subjective wellbeing (β = 0.168), organizational orientation: hierarchy-focused (β = 0.216), and duration of employment (β = 0.283) were positively associated with affective organizational commitment, while organizational orientation: ambivalent (β = −0.282) was negatively associated with affective commitment. In the public sector subsample, Agreeableness (β = 0.128), subjective wellbeing (β = 0.206), organizational orientation: hierarchy-focused (β = 0.124), and duration of employment (β = 0.311) were significant positive predictors of affective organizational commitment, while organizational orientation: ambivalent (β = −0.144) showed a negative relationship.

Table 4 presents the regression results for the continuance of organizational commitment across the full sample, as well as the private and public sector subsamples, highlighting the explained variance and significant predictors in each group. For the full sample, the model explained 18.8% of the variance in continuance organizational commitment (*R*^2^ = 0.188), with a statistically significant result, *F*_(13, 1, 036)_ = 18.492, *p* < 0.001. In the private sector subsample, the model accounted for 21.7% of the variance in continuance organizational commitment (*R*^2^ = 0.217), with a statistically significant result, *F*_(13, 489)_ = 10.416, *p* < 0.001. For the public sector subsample, the model explained 20.3% of the variance in continuance organizational commitment (*R*^2^ = 0.203), and the model was statistically significant, *F*_(13, 533)_ = 10.436, *p* < 0.001.

**Table 4:** Regression results for continuance organizational commitment in full, private, and public sector sample.

	**Full sample**					**Private sector**					**Public sector**				
	***B***	**SE**	β	**95% CI LB**	**95% CI UB**	***B***	**SE**	β	**95% CI LB**	**95% CI UB**	***B***	**SE**	β	**95% CI LB**	**95% CI UB**
Con.	2.316	1.465		−0.559	5.191	2.627	2.390		−2.069	7.322	2.438	1.872		−1.240	6.116
HH	0.026	0.015	0.056	−0.004	0.056	0.027	0.022	0.061	−0.016	0.070	0.024	0.022	0.049	−0.019	0.067
E	0.044	0.013	0.094	0.018	0.070	0.019	0.019	0.040	−0.019	0.057	0.076	0.019	0.159	0.038	0.113
Ex	0.001	0.019	0.002	−0.036	0.038	−0.003	0.030	−0.006	−0.063	0.056	−0.011	0.025	−0.020	−0.059	0.038
A	0.032	0.015	0.065	0.002	0.063	0.012	0.023	0.023	−0.033	0.057	0.054	0.021	0.108	0.013	0.095
C	0.007	0.018	0.014	−0.028	0.041	−0.018	0.026	−0.035	−0.069	0.033	0.026	0.025	0.054	−0.022	0.074
O	−0.055	0.012	−0.133	−0.079	−0.031	−0.071	0.017	−0.178	−0.105	−0.037	−0.042	0.018	−0.098	−0.077	−0.006
SWB	0.036	0.018	0.068	0.001	0.070	0.081	0.026	0.158	0.029	0.132	−0.003	0.024	−0.006	−0.051	0.044
HOO	0.066	0.018	0.131	0.030	0.102	0.100	0.027	0.199	0.047	0.154	0.035	0.025	0.068	−0.014	0.084
AOO	0.042	0.014	0.114	0.014	0.070	0.036	0.023	0.096	−0.009	0.081	0.039	0.019	0.107	0.001	0.077
IOO	0.036	0.015	0.084	0.006	0.066	0.016	0.021	0.041	−0.024	0.057	0.068	0.023	0.141	0.022	0.113
PEU	0.095	0.022	0.145	0.053	0.137	0.110	0.030	0.177	0.052	0.168	0.079	0.032	0.114	0.016	0.141
ED	0.085	0.008	0.306	0.069	0.102	0.103	0.013	0.351	0.077	0.129	0.067	0.011	0.251	0.045	0.089
IS	0.191	0.116	0.049	−0.036	0.417	0.158	0.165	0.040	−0.166	0.483	0.152	0.162	0.039	−0.166	0.470
*F*	18.492					10.416					10.436				
*df*	13,1,036					13,489					13,533				
*p*	0.000					0.000					0.000				
*R*	0.434					0.466					0.450				
*R^2^*	0.188					0.217					0.203				

HH, Honesty-Humility; E, Emotionality; Ex, Extraversion; A, Agreeableness; C, Conscientiousness; O, Openness to Experiences; SWB, Subjective Well-being; HOO, Organizational Orientation: Hierarchy-focused; AOO, Organizational Orientation: Ambivalent; IOO, Organizational Orientation: Indifferent; PEU, Perceived Employment Uncertainty; ED, Duration of Employment; IS, Degree of Income Satisfaction; B, unstandardized regression coefficient; SE, standard error; β, standardized regression coefficient; 95% CI LB, lower bound of the 95% confidence interval; 95% CI UB, upper bound of the 95% confidence interval.

For the full sample, emotionality (β = 0.094), openness to experiences (β = −0.133), perceived employment uncertainty (β = 0.145), and duration of employment (β = 0.306) were significant predictors of continuance organizational commitment, with positive associations except for openness to experiences, which was negatively associated. In the private sector subsample, subjective wellbeing (β = 0.158), organizational orientation: hierarchy-focused (β = 0.199), perceived employment uncertainty (β = 0.177), and duration of employment (β = 0.351) were positively associated with continuance organizational commitment. In the public sector subsample, emotionality (β = 0.159), agreeableness (β = 0.108), openness to experiences (β = −0.098), organizational orientation: indifferent (β = 0.141), perceived employment uncertainty (β = 0.114), and duration of employment (β = 0.251) were significant predictors of continuance organizational commitment.

Table 5 presents the regression results for normative organizational commitment across the full sample, as well as the private and public sector subsamples, showing the explained variance and the significant predictors in each group. For the full sample, the model explained 28.5% of the variance in normative organizational commitment (*R*^2^ = 0.285), with a statistically significant result, *F*_(13, 1, 036)_ = 31.841, *p* < 0.001. In the private sector subsample, the model accounted for 41.5% of the variance in normative organizational commitment (*R*^2^ = 0.415), with a statistically significant result, *F*_(13, 489)_ = 26.720, *p* < 0.001. For the public sector subsample, the model explained 25.3% of the variance in normative organizational commitment (*R*^2^ = 0.253), and the model was statistically significant, *F*_(13, 533)_ = 13.880, *p* < 0.001.

**Table 5:** Regression results for normative organizational commitment in full, private, and public sector sample.

	**Full sample**					**Private sector**					**Public sector**				
	***B***	**SE**	β	**95% CI LB**	**95% CI UB**	***B***	**SE**	β	**95% CI LB**	**95% CI UB**	***B***	**SE**	β	**95% CI LB**	**95% CI UB**
Con.	−2.959	2.552		−7.967	2.048	7.253	3.799		−0.211	14.717	−8.024	3.364		−14.632	−1.416
HH	0.103	0.027	0.119	0.051	0.155	0.117	0.035	0.144	0.049	0.186	0.067	0.039	0.074	−0.010	0.144
E	0.108	0.023	0.124	0.062	0.154	0.042	0.031	0.049	−0.019	0.102	0.146	0.034	0.165	0.078	0.214
Ex	0.022	0.033	0.022	−0.043	0.086	−0.055	0.048	−0.055	−0.150	0.039	0.034	0.044	0.035	−0.052	0.121
A	0.173	0.027	0.186	0.120	0.225	0.090	0.036	0.097	0.019	0.161	0.243	0.038	0.262	0.169	0.317
C	−0.058	0.031	−0.063	−0.118	0.002	−0.140	0.041	−0.150	−0.221	−0.060	0.018	0.044	0.020	−0.069	0.105
O	−0.051	0.021	−0.067	−0.093	−0.009	−0.033	0.027	−0.046	−0.087	0.020	−0.068	0.032	−0.087	−0.132	−0.005
SWB	0.158	0.031	0.164	0.098	0.218	0.221	0.042	0.235	0.138	0.303	0.110	0.043	0.113	0.025	0.196
HOO	0.187	0.032	0.199	0.125	0.249	0.236	0.043	0.254	0.152	0.321	0.110	0.045	0.115	0.022	0.197
AOO	−0.017	0.025	−0.024	−0.066	0.033	−0.130	0.036	−0.189	−0.201	−0.058	0.068	0.035	0.100	0.000	0.136
IOO	−0.082	0.027	−0.102	−0.134	−0.030	−0.118	0.033	−0.164	−0.182	−0.054	−0.015	0.042	−0.017	−0.097	0.067
PEU	0.001	0.037	0.001	−0.073	0.074	−0.028	0.047	−0.024	−0.120	0.064	0.021	0.057	0.017	−0.091	0.133
ED	0.161	0.015	0.313	0.133	0.190	0.167	0.021	0.311	0.126	0.208	0.156	0.020	0.313	0.116	0.195
IS	0.608	0.201	0.083	0.213	1.003	0.254	0.263	0.035	−0.263	0.770	0.893	0.291	0.123	0.322	1.464
*F*	31.841					26.720					13.880				
*df*	13,1,036					13,489					13,533				
*p*	0.000					0.000					0.000				
*R*	0.534					0.644					0.503				
*R^2^*	0.285					0.415					0.253				

HH, Honesty-Humility; E, Emotionality; Ex, Extraversion; A, Agreeableness; C, Conscientiousness; O, Openness to Experiences; SWB, Subjective Well-being; HOO, Organizational Orientation: Hierarchy-focused; AOO, Organizational Orientation: Ambivalent; IOO, Organizational Orientation: Indifferent; PEU, Perceived Employment Uncertainty; ED, Duration of Employment; IS, Degree of Income Satisfaction; B, unstandardized regression coefficient; SE, standard error; β, standardized regression coefficient; 95% CI LB, lower bound of the 95% confidence interval; 95% CI UB, upper bound of the 95% confidence interval.

For the full sample, honesty–humility (β = 0.119), emotionality (β = 0.124), agreeableness (β = 0.186), subjective wellbeing (β = 0.164), organizational orientation: hierarchy-focused (β = 0.199), and duration of employment (β = 0.313) were positively associated with normative organizational commitment, while organizational orientation: indifferent (β = −0.102) showed a negative relationship. In the private sector subsample, honesty–humility (β = 09.144), agreeableness (β = 0.097), subjective wellbeing (β = 0.235), organizational orientation: hierarchy-focused (β = 0.254), and duration of employment (β = 0.311) were positively associated with normative organizational commitment, while organizational orientation: ambivalent (β = −0.189) and organizational orientation: indifferent (β = −0.164) were negatively associated. In the public sector subsample, emotionality (β = 0.165), agreeableness (β = 0.262), subjective wellbeing (β = 0.113), organizational orientation: hierarchy-focused (β = 0.115), and duration of employment (β = 0.313) were significant positive predictors of normative organizational commitment.

The final set of analyses aimed to determine whether employees in the private sector could be distinguished from public sector employees based on affective, normative, and continuance organizational commitment (Table 6). Since the analysis of variance uses categorical independent variables and continuous dependent variables, and discriminant analysis utilizes continuous independent variables and a categorical dependent variable, a canonical discriminant analysis was performed. This analysis constructs a discriminant function using linear combinations of predictor variables to best differentiate between groups. The discriminant analysis also examines how individual variables contribute to group separation and the extent of their influence.

**Table 6:** Chi-square and functions at group centroids of the canonical discriminant functions.

**Wilks' Lambda**	**χ^2^**	***p***	**Function**	
0.956	50.263	0.000	Employees in the private sector	0.216
			Employees in the public sector	−0.211

Wilks' lambda, measure of the discriminant function's ability to differentiate between groups; Chi-square, test statistic for the significance of the discriminant function; p, significance level of the test; function, group centroids, representing the average score for each group (private and public sector).

The values of the group centroids (average discriminant scores for each group) range from −0.211 for employees in the public sector to 0.216 for employees in the private sector. The discriminant function was performed for 13 factors, Wilks' lambda = 0.956, χ^2^ = 5.263, *p* < 0.01 (Table 7).

**Table 7:** Means and structure matrix of canonical discriminant functions.

	**Employees in the private sector**	**Employees in the public sector**	***F***	***p***	**Stand. Can. Disc. Func. Coef**.
	***M***	***M***			
Affective commitment	15.2172	14.8298	3.126	0.077	−0.660
Continuance commitment	12.6145	13.9579	9.977	0.040	−1.036
Normative commitment	21.0700	19.5439	22.379	0.000	1.545

F, F-statistic indicating the significance of the difference between group means; p, significance level of the F-test; Stand. Can. Disc. Func. Coef., standardized canonical discriminant function coefficient representing the variable's contribution to the discriminant function.

Based on the data shown in Table 7, it can be concluded that employees in the private sector are characterized by a high level of normative organizational commitment, but low continuance commitment. On the other hand, employees in the public sector show the opposite results—they are characterized by high continuance, but low normative commitment.

## 4 Discussion

Contemporary trade, business, and economics trends suggest that the analysis and interpretation of organizational behavior increasingly need to adopt an ecological level of analysis. This approach emphasizes that neither the individual, with their internal traits, nor the organization as an independent entity can be the sole analysis unit. Instead, the focus should be on the dynamic interactions between individuals and their broader organizational and environmental contexts (Haslam, 2004; Haslam et al., 2010). Thus, in this study, in addition to the employees' personality traits of the employees, the focus was also on the variables of the wider socioeconomic context which refers to job certainty–uncertainty, but also to the sector (private- and state-owned sectors) in which the employees are active. The results of our study have indicated that the broad set of predictors, which consist of personality traits according to the HEXACO model, the organizational orientations of employees, perceived employment insecurity, subjective wellbeing, duration of employment, and income satisfaction are the best predictors of affective commitment (32.9%). In comparison, the percentage of the explained variability is somewhat lower when it comes to normative (28.7%), lowest when it comes to continuance commitment (19.2%).

The traits that emerged as most significant for explaining affective commitment include honesty, emotionality, agreeableness, and openness to experience, with the latter showing a negative partial contribution, indicating an inverse relationship with organizational commitment. Similar studies (Basnet and Regmi, 2019; Lee et al., 2020) have found that conscientiousness positively contributes to affective commitment. However, openness to experience has also been shown to have a statistically significant but negative contribution to affective commitment, as found by Choi et al. (2015) and Cui (2010). This suggests that higher levels of openness may be associated with decreased affective commitment to the organization. Several authors (Maertz and Griffeth, 2004; Raman et al., 2016; Salgado, 2002; Zimmerman, 2008) have also highlighted the link between openness to new experiences and a greater tendency to change workplaces.

Employees focused on advancing within the organizational hierarchy tend to exhibit higher organizational commitment, while ambivalent and indifferent organizational orientations show a negative partial contribution to organizational commitment. Weng and McElroy (2010) found that career growth positively influences commitment, particularly affective commitment, and Liu et al. (2020) similarly observed that organizational commitment fosters career advancement. Mathieu and Zajac (1990) concluded that organizational commitment, especially affective commitment, is stronger among individuals with professional advancement aspirations, high achievement motivation, and efficiency in environments that offer career progression. Todorović (2015) also identified a positive correlation between organizational commitment and employees' orientation toward career advancement while confirming a negative correlation with ambivalent and indifferent organizational orientations.

Perceived risk of job loss and difficult working conditions can undermine employees' subjective evaluations of their sense of belonging to an organization, negatively impacting organizational commitment (Miller et al., 2008; Obschonka and Silbereisen, 2015; Pinquart and Silbereisen, 2008). This aligns with our findings that highlight the positive association between continuance commitment and perceived employment uncertainty—employees facing a heightened perception of risk tend to seek stability by showing a stronger inclination to stay with their current organization. Additionally, duration of employment emerges as a significant variable in explaining organizational commitment, as work experience contributes positively to all aspects of commitment (affective, normative, and continuance). A notable finding is the positive contribution of subjective wellbeing to the variability in organizational commitment, which is consistent with results from similar studies (Caillier, 2013; Flint et al., 2013; Herrera and Heras-Rosas, 2021; Kundi et al., 2021; Salgado et al., 2019; Vanaki and Vagharseyyedin, 2009).

Furthermore, various studies have identified differences in work attitudes and organizational commitment between private and public sector employees (Boukamcha, 2022; Bullock et al., 2015; Crewson, 1997; Kumari and Pandey, 2011), and our findings support these differences. The regression results for public sector employees explain 51.8% of the variability in organizational commitment, compared to 39.2% for private sector employees, though both models are significant. An interesting finding is the better predictive power of the model for individual aspects of commitment—affective, normative, and continuance—in the private sector subsample, with evident, though not drastic, differences in partial contributions to criterion variability.

Hansen and Kjeldsen (2018) emphasize the importance of not only studying simple sectoral differences but also including relevant individual and organizational-level variables that explain the mechanisms behind such differences. The results of the canonical discriminant analysis confirmed these differences, particularly in the stronger expression of both normative and affective commitment among private sector employees, aligning with Genevičiute-Janone (2013), who found that private sector employees tend to exhibit higher levels of both affective and normative commitment. However, our findings diverge in showing a stronger manifestation of continuance commitment in the public sector.

Continuance commitment reflects employees' awareness of the cost of leaving an organization, such as lost investments and career advancements, which is more pronounced in the public sector, as confirmed by O'Neill et al. (2009). Crewson (1997) found that private sector employees tend to value reward more highly, while Boukamcha (2022) and Lyons et al. (2006) highlight that public sector employees often exhibit lower organizational commitment due to management styles focused on processes rather than outcomes. Additionally, public employees frequently feel that their personal goals and values do not align with those of their organization, contributing to lower levels of commitment.

Broader socioeconomic factors, particularly in transition economies such as Serbia, play an important role in the stronger manifestation of continuance commitment in the public sector. Public employment often provides a stable source of job security compared to the private sector, where economic instability and market fluctuations create greater uncertainty (Larimo and Arslan, 2013; Marković et al., 2011). Serbia's economic transition, characterized by high unemployment, privatization, and restructuring, has led many to view public sector jobs as safer, even though they may offer fewer opportunities for growth and innovation. The public sector is often perceived as a stable employer linked to the state, providing a safety net in times of economic uncertainty, which encourages employees to remain in their positions due to the perceived costs of leaving, such as the loss of benefits and financial stability (Todorović et al., 2017b). This reflects a continuation of Serbia's socialist past, where public roles were associated with stability and social welfare. In contrast, the private sector, influenced by multinational corporations and competitive market dynamics, attracts employees driven by career advancement and higher reward (Pavlović et al., 2022). This socioeconomic divide helps explain the differing levels of commitment, with public sector employees prioritizing job security and private sector employees seeking professional growth and innovation. Understanding these dynamics is essential, as the success of modern organizations in such contexts increasingly relies on adaptable and responsive human resources.

This study has limitations, which should be considered when interpreting the results. First, the cross-sectional design precludes any conclusions regarding causality, as the relationships observed between variables may not capture the directionality of their influence. Longitudinal studies would provide a more robust understanding of how organizational commitment evolves over time about the predictors examined. Second, self-reported data were used, raising the possibility of response biases such as social desirability, which could have influenced participants' assessments of commitment and related factors. Finally, while the socioecological model offers a broad framework, its scope makes it difficult to include all relevant variables. This study did not address leadership, a crucial factor influencing organizational commitment. Future research should explore how leadership styles interact with other socioecological factors to provide a more complete understanding of organizational commitment. Future research could also benefit from utilizing structural equation modeling to further explore the relationships between the variables. SEM offers a comprehensive approach to analyzing both direct and indirect effects, which could provide additional insights into the complexity of these relationships. All respondents in the study were from Serbia, a country whose socioeconomic and organizational landscape is characterized by a mix of private and public enterprises. This regional context, marked by a transitional economy, influences organizational culture and employee behavior, distinguishing it from more established market economies. When interpreting the findings and their applicability to other settings, these specifics should be considered.

## 5 Conclusion

In conclusion, this study underscores the complex interplay between individual, organizational, and socioeconomic factors in shaping organizational commitment, particularly in the context of a transitioning economy such as Serbia. By utilizing the socioecological model, we were able to provide a nuanced understanding of how personality traits, organizational orientations, subjective wellbeing, and employment uncertainty contribute to commitment across the public and private sectors. The findings emphasize the need for tailored organizational strategies that address both internal employee factors and external socioeconomic pressures.

This study offers both practical and theoretical contributions to the fields of organizational behavior and human resource management. Theoretically, it enriches the understanding of how individual personality traits, organizational orientations, subjective wellbeing, and perceived employment uncertainty collectively could potentially influence organizational commitment. Integrating the socioecological model into the analysis, the study contributes to a more comprehensive framework for examining organizational behavior, especially in transitional economies such as Serbia, where economic and social factors exert significant pressure on employee commitment. Practically, the findings have implications for organizational leaders and policymakers. In the public sector, where continuance commitment is prominent, efforts to enhance perceived organizational support and reduce employment uncertainty may help foster stronger affective and normative commitment. In the private sector, initiatives promoting career advancement and recognition may strengthen overall commitment. Moreover, the study highlights the importance of tailoring human resource strategies to the unique socioeconomic contexts in which organizations operate, ensuring that policies are aligned with employees' values, motivations, and external pressures.

## Data Availability

The original contributions presented in the study are included in the article/supplementary material, further inquiries can be directed to the corresponding author.
